# ER stress and UPR activation in glioblastoma: identification of a noncanonical PERK mechanism regulating GBM stem cells through SOX2 modulation

**DOI:** 10.1038/s41419-019-1934-1

**Published:** 2019-09-18

**Authors:** Natalia M. Peñaranda-Fajardo, Coby Meijer, Yuanke Liang, Bianca M. Dijkstra, Raul Aguirre-Gamboa, Wilfred F. A. den Dunnen, Frank A. E. Kruyt

**Affiliations:** 1 Department of Medical Oncology, University of Groningen, University Medical Center Groningen, Groningen, Netherlands; 2 Department of Neurosurgery, University of Groningen, University Medical Center Groningen, Groningen, Netherlands; 3 Department of Genetics, University of Groningen, University Medical Center Groningen, Groningen, Netherlands; 4 Department of Pathology, University of Groningen, University Medical Center Groningen, Groningen, Netherlands

**Keywords:** Cancer stem cells, CNS cancer

## Abstract

Patients with aggressive brain tumors, named glioblastoma multiforme (GBM), have a poor prognoses. Here we explored if the ER stress/unfolded protein response (UPR) is involved in the pathophysiology of GBM and may provide novel therapeutic targets. Immunohistochemical analyses of a tissue microarray containing primary GBM specimens showed strong variability in expression of the UPR markers GRP78/BiP, XBP1, and ATF4. Interestingly, high ATF4 expression was associated with poor overall survival suggesting involvement of PERK signaling in GBM progression. In vitro experiments using patient-derived neurospheres, enriched for GBM stem cells (GSCs), showed high sensitivity for the ER stressor thapsigargin (Tg) mainly via PERK signaling. In contrast, neurospheres-derived differentiated GBM cells were less sensitive likely due to lower UPR activity as indicated by comparative transcriptional profiling. Tg and Tunicamycin strongly reduced neurosphere forming ability of GSCs that was linked with potent PERK-dependent downregulation of SOX2 protein. Interestingly, SOX2 downregulation occurred directly via PERK, not requiring downstream activation of the PERK-UPR pathway. Moreover, PERK inactivation resulted in aberrant serum-induced differentiation of GBM neurospheres accompanied by persistent SOX2 expression, delayed upregulation of GFAP and reduced cell adherence. In conclusion, we provide evidence that PERK signaling contributes to the prognoses of primary GBM patients and identified PERK as a novel regulator of SOX2 expression and GSC differentiation. The role of PERK appeared to be pleiotropic involving UPR-dependent, as well as novel identified noncanonical mechanisms regulating SOX2. ER stress and PERK modulation appear to provide promising therapeutic targets for therapy in GBM.

## Introduction

Glioblastoma multiforme (GBM) is the most prevalent and lethal brain tumor in adults^1^. Surgery and chemo-radiotherapy lead to an expected median survival for newly diagnosed primary GBM of only 12–15 months and a 5-year survival rate of <5%^2^. Poor prognosis is caused by therapy resistance and high infiltrative growth of GBM, making complete resection impossible. Oncogenic driver mutations have been identified in GBM that affect retinoblastoma, p53 and receptor tyrosine kinase signaling, but targeting these pathways has not yet resulted in effective therapy^3,4^. Transcriptional profiling has identified several subtypes, named proneural (PN), classic, and mesenchymal (MES) GBM. The PN and MES subtypes appear most distinct and MES GBM being most aggressive with worst prognosis^5^. Currently, the *isocitrate dehydrogenase* (IDH) gene mutational status and methylation status of the MGMT promoter are used as prognostic markers in GBM^6^.

GBM tumors are cellular heterogeneous. GBM stem cells (GSCs) have been identified that possess self-renewal and differentiation ability, and are considered drivers of GBM growth, therapy resistance and relapse of disease^7,8^. Novel treatments that effectively target GSCs have been deemed essential for improving the prognosis of patients. In the current study we explored if ER stress and the unfolded protein response (UPR) affect GSCs and may provide novel targets for therapy.

The UPR is an essential adaptive mechanism that promotes cell survival under a variety of cell intrinsic and extrinsic adverse conditions including oncogenesis, hypoxia, glucose deprivation, and chemotherapy^9,10^. These conditions impact the biosynthetic demand and the correct production of proteins in the ER leading to UPR activation. The UPR attempts to restore protein homeostasis by halting protein production, enhancing protein folding capacity, and increasing protein degradation in order to facilitate cell survival, however, switches to cell death activation when damage is overwhelming.

Binding immunoglobulin protein/78 kDa glucose-regulated protein (BiP/GRP78) is a chaperone in the ER lumen and a central sensor for ER stress. Upon stress BiP/GRP78 is released from three ER-transmembrane proteins, RNA-dependent protein kinase-like ER kinase (PERK), inositol-requiring protein α (IRE1α), and activating transcription factor 6 (ATF6), leading to the activation of three distinct but partially functionally overlapping signaling pathways^11^. Through dimerization and auto-phosphorylation PERK activates the eukaryotic translation initiation factor 2α (eIF2α) leading to attenuation of global protein translation while specific mRNAs are translated, such as activating transcription factor 4 (ATF4). IRE1α oligomerization and auto-phosphorylation results in activation of its endoribonuclease activity and subsequent splicing of the X-box binding protein 1 (XBP1) mRNA yielding the transcription factor XBP1s. ATF6 undergoes cleavage in the Golgi and the ATF6f cleavage product also acts as a transcription factor. Subsequently, these transcription factors orchestrate the UPR including activation of the apoptosis transcription factor C/EBP-homologous protein (CHOP), when stress is overwhelming^12^.

The UPR also plays an important role in cancer and contributes to resistance to chemotherapeutics^13,14^. Notably, the UPR has been linked with reprogramming gene expression during tumor development and with the regulation of stem cell properties in both normal and malignant stem cells^15,16^. Promising novel therapeutic strategies have been developed to aggravate pre-existing (chronic) ER stress conditions in tumor cells by either increasing ER stress or inhibiting the UPR adaptive survival responses^13,17^.

In GBM chronic activation of the UPR has been reported evidenced by elevated BiP/GRP78 expression^18,19^. UPR inhibition was shown to sensitize for temozolomide, whereas the activity of for example radiotherapy was dependent on UPR-induced cell death^18,20^. Moreover, the UPR has been implicated in GBM growth and progression although its role in GSC maintenance remains elusive^21^.

Here we provide evidence that activation of the PERK branch of the UPR is involved in GBM prognoses by immunohistochemical analyses of UPR biomarkers in primary GBM specimens on a tissue microarray (TMA). Using GBM patient-derived neurospheres, known to contain GSCs and representing better the original tumor^22,23^, we found that GSCs are highly sensitive for ER stress. A key role for PERK in regulating ER stress-dependent self-renewal and differentiation of GSCs was found involving a novel noncanonical function that regulates SOX2 protein expression.

## Results

### BiP/GRP78, XBP1, and ATF4 expression in GBM TMA

**Fig. 1:**
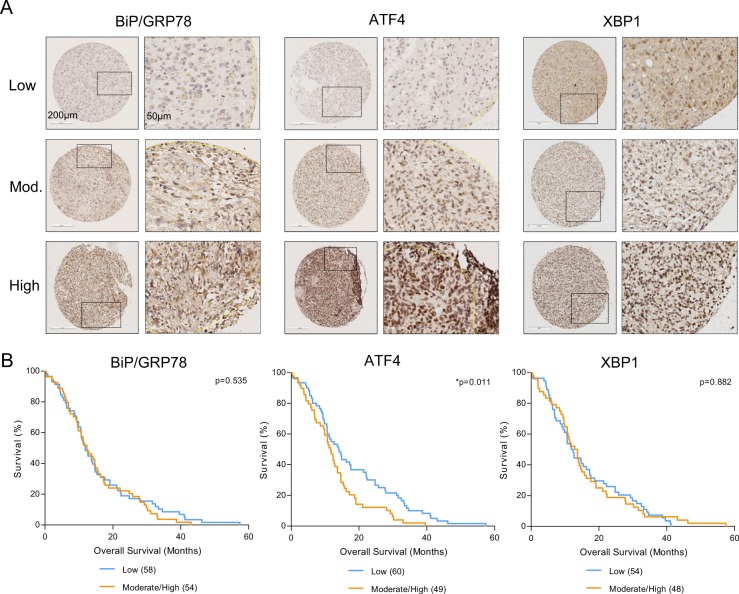
UPR marker expression in primary GBM specimens show correlation between ATF4 levels and overall survival. **a** Representative staining patterns of BiP/GRP78, ATF4, and XBP1 protein expression (low, moderate (mod.), and high) in GBM specimen cores on TMA. White scale bar is 200 µm. Enlarged boxed inserts are also depicted with scale bar of 50 µm. **b** Kaplan–Meier curves of OS related to the expression of low and moderate)/high expression of BiP/GRP78, ATF4, and nuclear XBP1. Number of patients in groups is indicated between brackets. Patients with ATF4^low^ expression had a better prognosis compared with ATF4^high^; for example, ATF4^low^ correlated with +/−35% OS vs 15% OS ATF4^high^ at 20 months post-surgery. *p-value < 0.05

A TMA containing specimens from 148 primary GBM patients (4 cores per patient) was used to examine expression of BiP/GRP78, ATF4, and XBP1. Main characteristics of patients are summarized in Table 1. BiP/GRP78 staining was cytoplasmic, ATF4 nuclear and XBP1 was localized both in cytoplasm and nucleus reflecting inactive and active splice variants, respectively (Fig. 1a). For XBP1 only nuclear staining was scored. Expression was classified according to the median staining score in low and moderate-high expressing groups. BiP/GRP78 was frequently co-expressed with ATF4 with a significant correlation factor of 0.217 and also ATF4 and XBP1 expression were positively correlated with a significant correlation factor of 0.203 (Supplementary Table 1). Interestingly, low ATF4 expression correlated with prolonged overall survival (OS), whereas BiP/GRP78 and XBP1 expression did not correlate with OS (Fig. 1b). Together, these findings suggest a link between the PERK branch of the UPR and prognosis of GBM patients.

**Table 1:** Patient characteristics

Number of primary GBM patients	148
Mean age at surgery in years (range)	61.9 (30.9–84.6)
Number >70 years (%)	29 (19.6)
Male sex (%)	94 (63.5)
Still alive (%)	6 (4.1)
Mean OS in months^a^ (range)	13.7 (0.2–57.4)

^a^OS = overall survival, time between date of surgery and documented date of death, living patients excluded

### ER stress sensitivity and UPR activation in GBM neurospheres

**Fig. 2:**
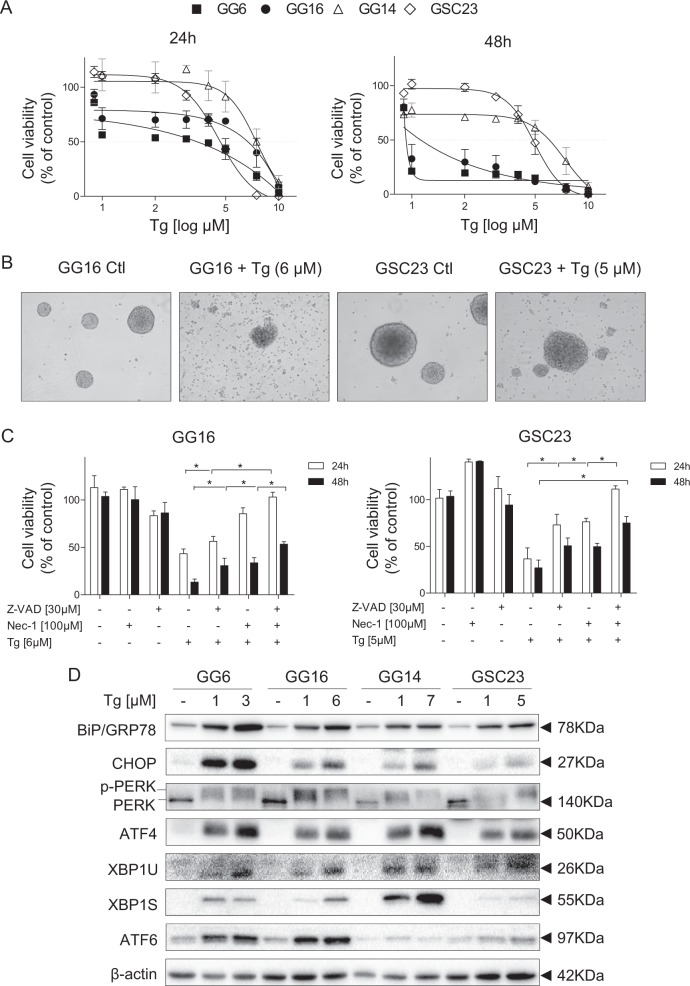
GBM neurospheres/GSCs are sensitive for Tg that is accompanied by UPR activation. **a** GBM Nsp cells were treated with different doses of Tg for 24 or 48 h. Cell viability was evaluated by MTS assays. Dose and time-dependent cytotoxicity was observed. **b** Preformed GG16 and GSC23 Nsp were exposed to Tg for 24 h. Phase contrast microscopy (×10) showed strong toxicity as indicated by Nsp disintegration and debris. **c** MTS assays of Nsp cells treated with combinations of Tg and the pan-caspase inhibitor Z-VAD, the RIPK1 inhibitor Nec-1 or with the inhibitors combined. Both apoptosis and necroptosis activation contribute to cytotoxicity. **d** Western blots showing Tg-induced activation of the UPR by increased BiP/GRP78 and CHOP levels. Tg activated all three UPR branches to varying extents in a cell specific way as indicated by PERK phosphorylation (upper band) and ATF4 expression, enhanced expression of XBP1S and ATF6. Error bars indicate standard deviations. *p-value < 0.05

To investigate this further, first we characterized a panel of GBM neurospheres for sensitivity to the well-known ER stress inducer Tg, a sarco/endoplasmic reticulum Ca^2+^ ATPase (SERCA) inhibitor. A dose-dependent reduction of cell viability was seen after 24 h treatment that became more pronounced after 48 h, particularly for MES GG6 and GG16 (Fig. 2a). Similar cytotoxicity profiles were seen upon exposure to another ER stress inducer, tunicamycin (Tm), although with no clear difference between PN (GG14 and GSC23) and MES subtypes (Supplementary Fig. 1A). Preformed GBM GG16 and GSC23 neurospheres were also sensitive for Tg indicated by sphere disintegration and increased levels of cellular debris (Fig. 2b).

Tg exposure was accompanied by caspase-3/7 activation in GG16 and GSC23 cells (Supplementary Fig. 2). The broad caspase and apoptosis inhibitor Z-VAD partially suppressed Tg cytotoxicity (Fig. 2c). The involvement of necroptosis was explored by inhibiting RIPK1 with necrostatin-1 (Nec-1) and showed also partial protection. Combined Z-VAD/Nec-1 treatment completely protected against Tg cytotoxicity after 24 h exposure, although cell viability decayed at 48 h treatment.

Tg treatment activated the UPR as indicated by strong induction of BiP/GRP78 in all GBM neurospheres at both a low (1 µM) and higher dose Tg (IC_50_) (Fig. 2d). Tg exposure induced PERK phosphorylation, represented by occurrence of a higher molecular weight band and accumulation of ATF4. IRE1α was also activated reflected by increased XBP1u expression and occurrence of XBP1s. ATF6 was already detected in untreated neurospheres and accumulated further after treatment. In parallel, concentration-dependent accumulation of CHOP was observed.

Thus, GBM neurospheres are highly sensitive to Tg, accompanied by variable activation of all three UPR branches leading to both apoptosis and necroptosis.

### Differentiation of GBM cells reduces thapsigargin sensitivity

**Fig. 3:**
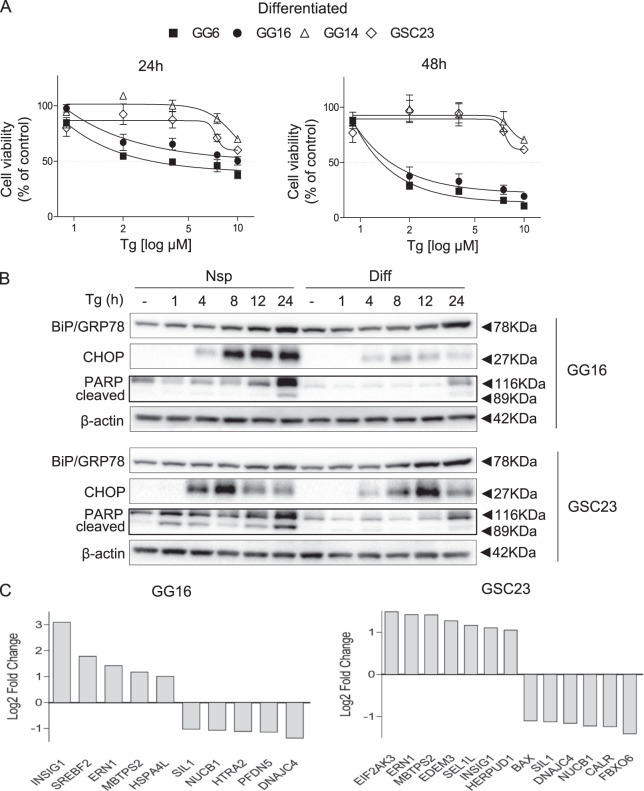
Differentiated GBM cells are less sensitive for Tg. **a** MTS assays showing Tg sensitivity of serum-differentiated neurospheres after 24 and 48 h treatment. Dose and time-dependent cytotoxicity was observed. **b** Western blots showing time-dependent activation of the UPR by Tg, represented by expression of BiP/GRP78, CHOP, and PARP cleavage. **c** RNA-Seq transcript analyses was used to compare the expression of UPR-related genes in GG16 and GSC23 neurospheres vs. differentiated counterparts. Overall the major UPR branches signals appeared to be higher expressed in neurospheres (see also Supplementary Fig. 3). Genes that have a Log2 Fold Change >1 an adjusted *p*-value ≤ 0.05 are depicted

Since tumors are heterogeneous in GSC/non-GSC composition we examined Tg sensitivity in serum-differentiated GBM neurospheres. Differentiated GBM cells were more resistant to Tg when compared with corresponding neurospheres (Fig. 3a and Table 2). Particularly differentiated PN GG14 and GSC23 cells appeared resistant also after prolonged Tg treatment. Analyses of Tg-induced UPR activation indicated a stronger increase of BiP/GRP78, CHOP expression and PARP cleavage in GG16 and GSC23 neurospheres compared with differentiated counterparts (Fig. 3b). UPR branch activation was also seen in differentiated cells, although PERK branch activation appeared reduced in differentiated GSC23 cells and also p-IRE1α levels differed in cells (Supplementary Fig. 3A).

**Table 2:** Tg-induced cytotoxicity in GBM neurospheres and differentiated (Diff.) counterparts measured by MTS

	Tg [µM]					
	IC_25_		IC_50_		IC_75_	
	24 h	48 h	24 h	48 h	24 h	48 h
GG6	0.9	0.9	3.5	0.9	6.9	1.0
Diff.GG6	1	0.9	4	1.2	^a^	2.2
GG16	2.8	^a^	6.6	1.1	8.8	2.3
Diff.GG16	1.3	1	^a^	1.3	^a^	2.8
GG14	6.2	^a^	7.5	7.4	8.7	7.9
Diff.GG14	9.3	8.8	^a^	^a^	^a^	^a^
GSC23	4.3	3.9	5.3	4.8	6.4	5.9
Diff.GSC23	6.9	7.6	^a^	^a^	^a^	^a^

^a^>10 µM Tg

To obtain molecular insight in differences in Tg sensitivity RNA sequencing was performed. Comparing transcript levels of genes involved in the UPR between neurospheres and differentiated GG16 and GSC23 cells revealed clear differences in UPR genes expression patterns (Supplementary Fig. 3B). Notably, the transcriptional levels of key players in the three UPR branches were elevated in neurospheres, including *endoplasmic reticulum to nucleus signaling 1* (*ERN1*, encoding IRE1α), *XBP1*, *EIF2AK3* (encoding PERK), and *ATF6*, whereas expression of the negative regulator of eIF2α, *protein phosphatase 1 regulatory subunit 15A* (*PPP1R15A*, encoding GADD34), was decreased. Focusing on genes with at least twofold difference in expression, five overlapping genes were identified in GG16 and GSC23 (Fig. 3c). Neurospheres showed increased expression of *ERN1*, *insulin-induced gene 1* (*INSIG1*), and *membrane-bound transcription factor peptidase site 2* (*MBTPS2*) and decreased expression of *SIL1 nucleotide exchange factor* (*SIL1*) and *Nucleobindin 1* (*NUCB1*). Overall, these findings suggest a link between elevated UPR activity in GBM neurospheres compared with differentiated cells and higher sensitivity for Tg in neurospheres.

### PERK branch mediates ER stress-induced cytotoxicity in GBM neurospheres

**Fig. 4:**
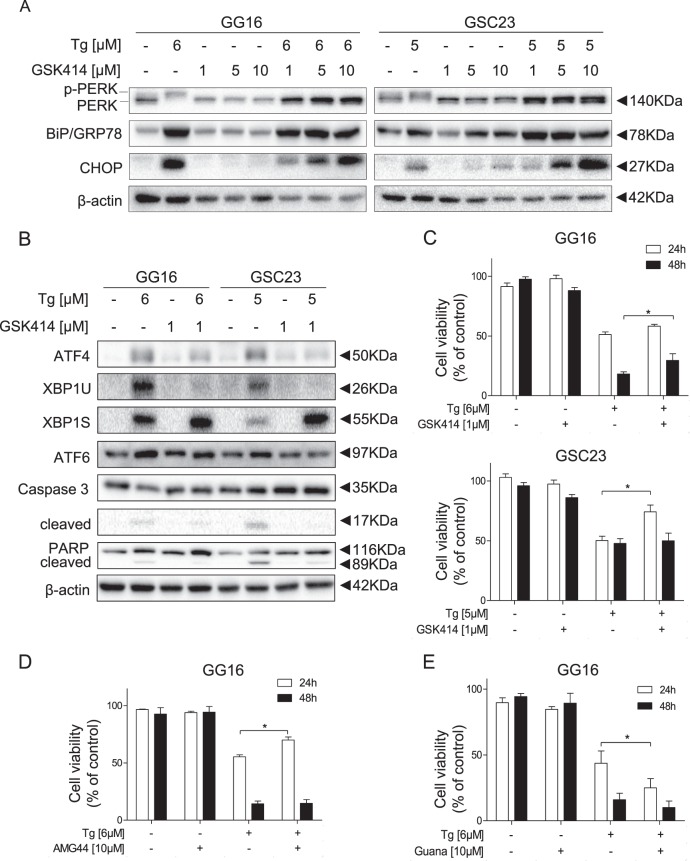
PERK mediates Tg-induced cytotoxicity in GBM neurospheres/GSCs. **a** Western blots showing the effect of varying concentrations PERK inhibitor GSK414 on PERK phosphorylation, BiP/GRP78, and CHOP expression in GG16 and GSC23 in absence or presence of Tg. Co-treatment with 1 µM GSK414 for 24 h showed potent PERK inhibition and reduction of CHOP. **b** GSK414 prevented Tg-induced ATF4 accumulation and caspase-3/PARP cleavage. Co-treatment with Tg and GSK414 hardly altered ATF6 levels whereas XBP1 processing was increased. **c** MTS assays showing PERK inhibition by GSK414 or **d** AMG44 suppressed Tg-induced cytotoxicity, whereas prolonged stimulation of PERK-eIF2α signaling by Guanabenz (Guana) enhances cytotoxicity (**e**). Error bars indicate standard deviations. *p-value < 0.05

The correlation found between ATF4 expression and overall patient survival led us to test if the PERK branch is involved in Tg cytotoxicity by using PERK inhibitor GSK2606414 (GSK414)^17,24^. GSK414 strongly reduced Tg-dependent induction of p-PERK, ATF4 and CHOP in GG16 and GSC23 cells; optimal inhibition was seen at 1 µM GSK414 since higher concentrations also led to increases in CHOP expression (Fig. 4a, b). PERK inhibition resulted in enhanced accumulation of XBP1s, probably as a compensatory mechanism (Fig. 4b). PERK inhibition suppressed Tg-induced cytotoxicity that was associated with decreased cleavage of caspase-3 and PARP (Fig. 4b, c). Another pharmacological PERK inhibitor, AMG44^25^, also effectively blocked PERK activation and showed similar suppression of Tg cytotoxicity in GG16 (Fig. 4d) and to a lesser extent in GSC23 (Supplementary Fig. 4). As an alternative approach we tested the effect of prolonged activation of the PERK branch on Tg cytotoxicity by employing the eIF2α phosphatase GADD34 inhibitor Guanabenz (Guana)^26^. Combined Tg/Guana treatment resulted in enhanced induction of phosphorylated eIF2α as well as CHOP accumulation, although to variable extents in a cell-dependent way (Supplementary Fig. 4C), and enhanced Tg cytotoxicity (Fig. 4e and Supplementary Fig. 4D).

The involvement of the other two UPR branches in mediating ER stress-induced cytotoxicity in the GBM neurospheres models was also evaluated. However, inhibition of the IRE1α/XBP1 or ATF6 branch by chemical inhibitors or shRNA-mediated gene silencing, respectively, did not affect Tg sensitivity (Supplementary Fig. 5A–D). Overall, these data indicate that the PERK branch mainly mediates Tg cytotoxicity in GBM neurospheres.

### Thapsigargin reduces neurosphere formation ability accompanied by SOX2 downregulation

**Fig. 5:**
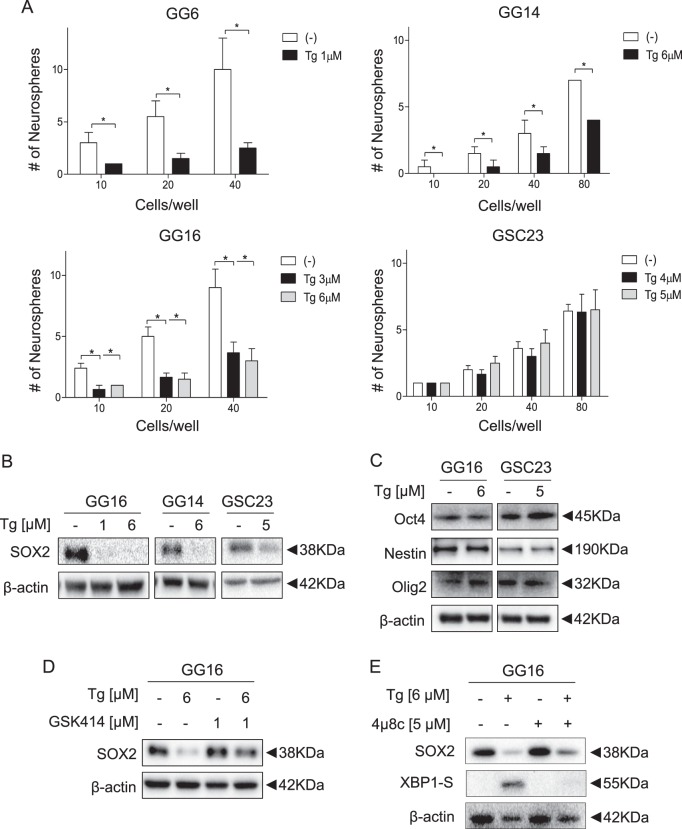
Tg reduces neurosphere formation capacity of GSCs that is associated with PERK-dependent downregulation of SOX2 expression. **a** Limiting dilution assays of GG6 and GG14 Nsp cells treated for 24 h with Tg IC_25_ concentrations lead to a potent reduction of neurospheres formation capacity. Treatment of GG16 and GSC23 Nsp with Tg IC_25_ and IC_50_ concentrations showed differential reduction of neurospheres formation capacity in GG16 but not in GSC23. **b** Western blots demonstrating a strong reduction of SOX2 expression upon Tg treatment, whereas protein expression of other GSC markers Oct4, Nestin, and Olig2 was not affected (**c**). **d** Western blots showing rescue of Tg-dependent reduction of SOX2 expression by GSK414 co-treatment. **e** No rescue of SOX2 expression was seen by cotreating with the IRE1α inhibitor 4µ8c. Error bars indicate standard deviations. *p-value < 0.05

According to the CSC hypothesis effective therapy should target GSCs^27^. Although GBM neurospheres represent undifferentiated GBM cells it should be noted that only a proportion of cells have self-renewal potential that is characteristic for GSCs. To examine the effect of Tg on self-renewal of GSCs limiting dilution neurosphere formation assays were performed. Tg resulted in a two- to fourfold reduction in neurosphere formation ability in GG6, GG14, and GG16, whereas in GSC23 no significant effects were seen (Fig. 5a). Tm similarly suppressed self-renewal in GG16 cells, but hardly in GSC23 (Supplementary Fig. 1B).

Next, we examined if Tg affects the expression of SOX2, a well-known stem cell transcription factor in neuronal stem cells and GSCs^28–30^. Interestingly, a robust decrease in SOX2 protein expression was observed upon 24 h Tg treatment. SOX2 decrease was particularly strong in GG16 and GG14 cells, detectable already at low Tg concentration (Fig. 5b). Weaker SOX2 downregulation was seen in GSC23 that may in part explain reduced Tg sensitivity. Notably, Tg did not affect the expression of other stem cell transcription factors such as OCT4 and Olig2 and the stem cell marker Nestin (Fig. 5c).

We proceeded by testing if the PERK branch mediates SOX2 downregulation. Tg combined with GSK414 potently suppressed SOX2 downregulation in GG16 cells, whereas inhibition of the IRE1/XBP1 branch did not have this effect (Fig. 5d, e). Tm also reduced SOX2 expression that was prevented by GSK414 (Supplementary Fig. 1C). Thus, ER stress leads specifically to downregulation of SOX2 expression via the PERK branch providing a molecular mechanism for GSC targeting.

### PERK regulates ER stress induced SOX2 downregulation

**Fig. 6:**
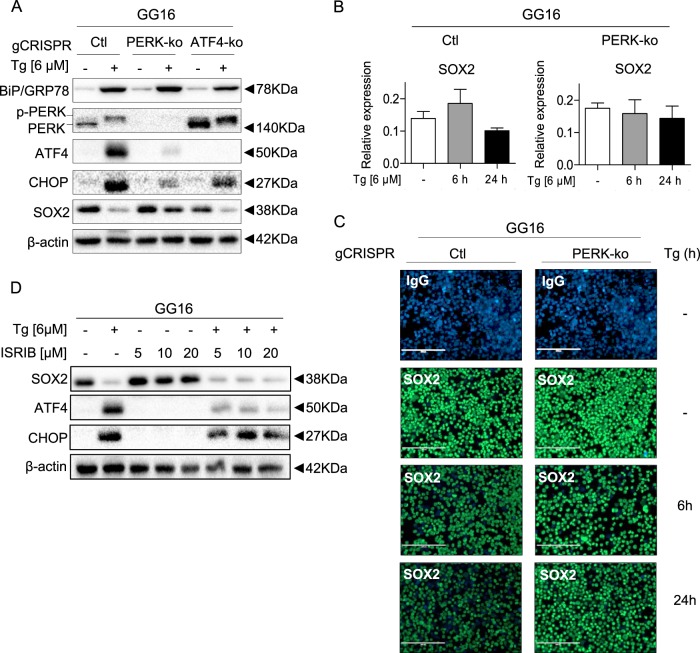
PERK directly regulates ER stress induced SOX2 downregulation in GSCs. **a** BiP/GRP78, PERK, ATF4, CHOP, and SOX2 expression in GG16-PERK or -ATF4 knockout (ko) cells treated with Tg, showing effective knockout of PERK and ATF4 and rescue of SOX2 downregulation in PERK-ko cells. **b** RT-qPCR analyses was performed to determine the effect of 6 and 24 h IC_50_ Tg exposure on the expression of SOX2 GG16-PERK-ko. **c** Representative IF images of SOX2 expression (green) in GG16 control and GG16-PERK-ko cells treated with(out) Tg for different times. Cells nuclei were stained with DAPI (blue). SOX2 levels remain high in PERK-ko cells. **d** SOX2, ATF4, and CHOP protein expression in GG16 neurospheres after treatment with Tg in absence/presence of eIF2α inhibitor ISRIB. Error bars indicate standard deviations. *p-value < 0.05

To explore in more detail how the PERK branch regulates SOX2 expression PERK and ATF4 knockouts were generated in GG16 cells using CRISPR/CAS9 genomic editing. Figure 6a, shows effective ablation of the *EIF2AK3* gene in GG16-PERK-ko cells indicated by the absence of PERK protein expression and almost absence of ATF4 induction after Tg exposure and strong reduction of CHOP accumulation. GG16-ATF4-ko cells showed normal Tg-induced PERK activation but complete absence of ATF4 induction illustrating effective *ATF4* ablation. Importantly, SOX2 downregulation was largely suppressed in GG16-PERK-ko cells, whereas in GG16-ATF4-ko cells potent SOX2 downregulation was observed similar to control GG16 cells. SOX2 downregulation occurred at the protein level since mRNA levels did not change significantly in GG16 control and PERK-ko cells (Fig. 6b).

To analyze SOX2 decay at the cellular level immunofluorescence microscopic analyses was performed showing abundant nuclear SOX2 protein expression in the large majority of GG16 control and GG16-PERK-ko cells. Tg treatment resulted in a strong general decrease in SOX2 expression in control cells that was already detectable 6 h post-treatment, decreasing further after 24 h and some cells showing complete loss of SOX2 expression (Fig. 6c). In contrast, GG16-PERK-ko cells showed only minor decrease in SOX2 levels.

The possible involvement of eIF2α in SOX2 downregulation was also investigated by using ISRIB, an inhibitor known to reverse eIF2α phosphorylation^31^. ISRIB potently reduced Tg-induced eIF2α-dependent activation of ATF4 expression and also reduced CHOP levels, however, did not affect Tg-dependent SOX2 downregulation (Fig. 6d). Since we already ruled out ATF4, these results show that SOX2 expression is regulated directly via PERK.

### PERK regulates SOX2 expression and differentiation of GBM neurospheres

**Fig. 7:**
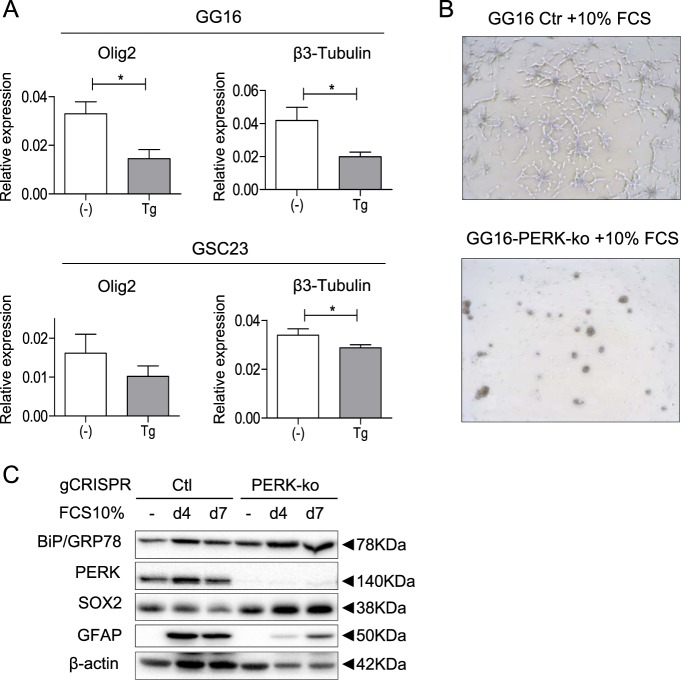
PERK regulates SOX2 expression during serum-induced differentiation of GBM neurospheres/GSCs. **a** RT-qPCR analyses was performed to determine the effect of 24 h IC_50_ Tg exposure on the expression of Olig2 and β3-Tubulin of GG16 cells. **b** Representative phase contrast microscopy images (×10) of GG16 control and GG16-PERK-ko cells after 7 days serum exposure. **c** Protein expression of BiP/GRP78, PERK, SOX2, and GFAP during serum differentiation of GG16-PERK-ko cells, showing aberrant differentiation in PERK-ko cells. Error bars indicate standard deviations. *p-value < 0.05

ER stress has been reported to induce differentiation of colon cancer stem cells^32^. To test if ER stress similarly would induce differentiation in GBM neurospheres mRNA levels of the astrocytic marker GFAP and the neuronal markers OLIG2 and b3-Tubulin were determined in GG16 and GSC23 neurospheres exposed to Tg. GFAP mRNA levels were hardly detectable by qRT-PCR (not shown), whereas OLIG2 and b3-Tubulin levels decreased, thus providing no evidence for Tg-induced differentiation (Fig. 7a).

We also studied the effect of PERK knockout on serum-induced differentiation of GG16 neurospheres. Interestingly, absence of PERK resulted in impaired cell adhesion compared with rapid adherence normally seen after serum addition as observed by microscopy (Fig. 7b). Moreover, western blots of serum-exposed GG16-PERK-ko neurosphere cells demonstrated an impaired time-dependent decrease in SOX2 expression in combination with reduced accumulation of GFAP, which is normally seen in serum-differentiated GG16 cells (Fig. 7c). Thus, PERK also regulates SOX2 expression during serum-induced differentiation and identifies PERK as an important mediator of GSC differentiation.

## Discussion

In this study, using both primary GBM specimens and GBM neurosphere models we examined the impact of ER stress and the UPR on patient prognoses and GSC viability and identified a novel role for PERK in GSCs self-renewal and differentiation.

Evaluation of UPR biomarker expression in primary GBM samples on TMA revealed that high levels of ATF4 is associated with poor prognosis in treatment naive patients. This suggests that low activity of the PERK/eIF2α/ATF4 branch is beneficial for prognosis. Our data are the first showing ATF4 protein expression in a large GBM patient dataset, and are in line with a recent report showing that low ATF4 transcript levels in the NIH Rembrandt Glioma database is associated with prolonged survival^33^. Although we did not find significant associations between BiP/GRP78 or XBP1 expression in the GBM specimens and OS, correlations were found between BiP/GRP78 and ATF4 expression and between ATF4 and XBP1, suggesting associations between ER stress and UPR activation. Others have reported prognostic relevance of BiP/GRP78 and IRE1/XBP1 in GBM, however, this was predominantly based on mRNA levels^34,35^. Of note, ATF4 is activated via eIF2α, a key player of the integrated stress response, able to respond to various micro-environmental stresses such as hypoxia, nutrient, glucose and amino acid shortage, involving a number of upstream kinases including PERK^36^. Therefore ATF4 accumulation is not solely the result of ER stress. ATF4 has been implicated in promoting angiogenesis, invasion and temozolomide resistance, which may explain poor prognoses in high ATF4 expressing GBM patients^37,38^.

Our in vitro studies showed that particularly GBM neurospheres were sensitive for Tg involving activation of the PERK branch. Neurospheres belonging to the MES subtype were more sensitive for Tg compared with PN neurospheres. Overall, Tm-induced ER stress had similar cytotoxic effects in these models, but differences between PN and MES GBMs were less obvious. Notably, a link between mesenchymal phenotype, increased UPR activity and ER stress sensitivity has been reported in breast cancer cells likely related to increased secretory activity^39^. Regardless of the subtype, GBM neurospheres showed Tg dose- and cell-dependent activation of all three UPR branches known to orchestrate an adaptive survival response^12,40^. Cytotoxicity was accompanied by CHOP accumulation, caspase-3/7 activation and PARP cleavage indicative of apoptosis, which was corroborated by decreased cytotoxicity upon co-administration with the pan-caspase inhibitor Z-VAD. Interestingly, Tg also induced necroptosis that could be inhibited by the RIPK1 inhibitor Nec-1. Necroptosis activation by ER stress has not been frequently reported. Saveljeva et al.^41^ found that Tm activates ligand-independent tumor necrosis factor receptor 1 (TNFR1)-mediated necroptosis in murine fibroblasts and TNFR1/RIPK1 inhibition induced a switch to apoptotic death. In the present study such a switch was not seen and combined blocking of apoptosis and necroptosis effectively suppressed Tg-induced cell death.

To mimic cellular heterogeneity in GBM, also serum-differentiated neurospheres were examined. Differentiated GBM cells appeared to be more resistant for Tg than undifferentiated counterparts, which was linked with reduced and altered UPR activation in a cell-dependent manner. The underlying causes require more in depth analysis. Regardless of that ER stress aggravation appears particularly promising for eradicating the stem cell compartment of GBM. Transcriptomic analyses of UPR-related genes provided mechanistic clues for differences in Tg sensitivity between neurospheres and differentiated cells. Neurospheres showed an overall increase in expression of the main ER stress sensors, likely reflecting higher UPR activity and a higher demand on protein quality control in undifferentiated GBM cells. Among the strongest upregulated genes were *INSIG1* and *MBTPS2* known to play a role in cholesterol metabolism and regulators of sterol regulatory element-binding proteins (SREBPs) including ATF6, and ERAD. *SIL1*, encoding a nucleotide exchange factor for BiP/GRP78 and *NUCB1*, a calcium binding protein involved in maintaining calcium homeostasis, were downregulated. Their precise relationship with ER stress sensitivity in neurospheres/GSCs remains to be explored.

Importantly, the present study shows that ER stress aggravation targets GSCs, considered key drivers of tumor growth, aggressiveness and therapy resistance in GBM^1,7,8^. ER stress induction by both Tg and Tm effectively reduced neurosphere formation in limiting dilution assays in three of the four GBM neurosphere models tested, indicating that ER stress suppresses self-renewal potential of GSCs. This is in accordance with the notion that UPR activity is necessary for stem cell maintenance, as was demonstrated for example in murine neural stem cells^16,42,43^. Tg treatment of GSC/neurospheres did not increase the expression of differentiation markers, which is different to the situation in colon cancer stem cells where ER stress induction has been reported to trigger differentiation and sensitization for chemotherapy^32^.

**Fig. 8:**
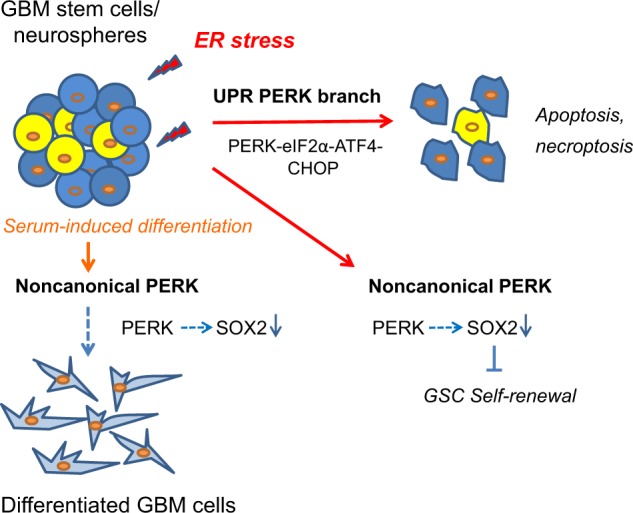
Proposed model for regular and noncanonical PERK-dependent signaling regulating ER stress cytotoxicity, self-renewal, and differentiation in GBM. Summarizing figure depicting the main findings of this study. The PERK branch of the UPR appears to be a main inducer of apoptotic and necroptotic cell death in GSC/neurospheres that suffer from ER stress-induced cytotoxicity. In parallel, ER stress, also at lower levels, activates noncanonical PERK signaling independent of the well-known downstream effectors eIF2α and ATF4. This mechanism is able to downregulate protein levels of the stem cell transcription factor SOX2 resulting in loss of stem cell potential. Also more physiological conditions such as serum-induced differentiation require noncanonical PERK signaling since genetic-depletion of PERK resulted in aberrant differentiation characterized with persistent SOX2 expression and impaired cell adherence. The molecular link between PERK and SOX2 modulation remains to be identified. See text for more details

We could associate loss of self-renewal capacity with a specific strong decrease in SOX2 protein expression, a well-known neural stem cell and GSC transcription factor^28–30^. This is in agreement with a recent study showing that SOX2 expression is reduced upon exposure to Tm in GBM neurospheres^44^. Interestingly, we found that PERK directly regulates SOX2 downregulation at the protein level, independent from eIF2α/ATF4 signaling, thus identifying a novel noncanonical function for PERK. SOX2 downregulation required the kinase function of PERK, since GSK414 was sufficient to prevent downregulation. Moreover, PERK-ko GBM neurospheres displayed aberrant serum-induced differentiation characterized by failure to downregulate SOX2 and disrupted upregulation of differentiation markers. This indicates that other yet unknown signals or perhaps more subtle alterations in protein-homeostasis associated with differentiation may activate PERK. Our findings, summarized in Fig. 8, add to the notion that PERK has additional functions. For example, a UPR-independent function for PERK has been reported by van Vliet et al. involving direct interaction between PERK and Filamin A and regulation of F-actin remodeling and calcium homeostasis^45^. We have not been able to detect direct SOX2-PERK protein interactions (not shown) and the identified noncanonical mechanisms of PERK remains to be further elucidated.

From a therapeutic standpoint we propose that ER stress aggravation in GBM cells is beneficial for patients since it will result in depletion of the GSC compartment. Although radiation and chemotherapy induce in part the UPR, the development of clinical safe ER stress inducers able to pass the blood-brain-barrier would be of great interest. On the other hand, we predict that the use of PERK inhibitors will potentially have great clinical value since it will impair the plasticity of GSCs making them less able to adapt to changes in the microenvironment and toward therapy. However, this remains to be studied further.

In conclusion, we found that the PERK pathway contributes to ER stress-induced cytotoxicity in GBM neurospheres and identified a noncanonical PERK-dependent mechanism that regulates GSC self-renewal and differentiation involving posttranscriptional regulation of SOX2 expression via an as yet unknown mechanism. The development of clinical applicable ER stress inducers and PERK inhibitors holds promise as therapeutic strategies in GBM.

## Materials and methods

### Tissue micro arrays

Tissue samples of glioma grade IV diagnosed adult patients without previous treatment or IDH mutations were collected from November 2005 to January 2016 at our institute for generating a TMA (4 cores per patient) as described by Conroy et al.^46^. All experiments using human tissue were conducted under the ‘Code of Conduct for dealing responsibly with human tissue in the context of health research’ published by the Federation of Dutch Medical Scientific Societies in 2011 (www.federa.org) and approved by the local ethics review board on behalf of the medical ethical committee (METC) of the UMCG (see Supplementary methods for more details).

### Immunohistochemistry

Staining for BiP/GRP78, XBP1, and ATF4 was performed according to standard protocols; positive and negative controls, including immunoglobulin class-matched controls (Diagnostics BV, Uithoorn, Netherlands) were used for each staining. For detailed description of staining, scoring and analysis (see Supplementary Methods and Supplementary Table 2). Images were digitalized using the C9600 NanoZoomer (Hamamatsu Photonics KK, Almere, Netherlands). Scoring of BiP/GRP78 and ATF4 was performed automatically by using the positive pixel count algorithm and software of Aperio Image Scope 12.3.3 (Leica Biosystems, Amsterdam, Netherlands). For evaluation of BiP/GRP78 and ATF4 staining the scores were divided into two groups according to the median in low and moderate-high expressing groups. XBP1 expression was determined by scoring nuclear staining intensity and percentage of positive cells by two independent observers (NP and CM) blinded for patient outcome and random samples were validated by a blinded expert pathologist (WFAvD) XBP1 expression was scored according to the immunoreactive score (IRS) that was divided into two groups according to the median into low and moderate-high staining groups. Overall survival (OS) was defined as the time between date of surgery and the documented date of death. Shapiro–Wilk normalization indicated no normal distributions of the staining, and therefore a nonparametric statistical method was performed for correlation analyses. Correlations between patients characteristics and UPR biomarker co-expression were tested using Spearman’s nonparametric correlation testing. Survival curves were calculated with the Kaplan–Meier method using the log-rank test after correction for age over 70 years old that is a confounding factor for survival. All tests were two-sided and a *p*-value of <0.05 was considered significant. Statistical analysis was performed by using the statistical software SPSS 23.0 (IBM SPSS, Armonk, New York, USA).

### Cell culture

The GBM neurospheres used in this study have been described before^29,47^ and were generated from surgical leftovers obtained from anonymous GBM patients after approval and following the ethical guidelines of the Medical Ethics Review Committee (METC) of the University Medical Center Groningen (UMCG). The patient-derived GBM neurospheres GG6, GG14, GG16, and GSC23 were cultured in neural stem cell medium (NSM) as previously described, GG6 and GG16 representing MES GBM, GG14 and GSC23, PN GBM^47^. GBM neurospheres were differentiated with 10% FCS culture medium^29^. The GBM cell line GSC23 was kindly provided by Krishna Bhat, PhD (Translational Molecular Pathology, Department of Pathology, MD Anderson Cancer Center, Texas University, USA). Cells were tested regularly by SSTR profiling and for mycoplasma.

### Cell viability and caspase activity assays

Cells were seeded in triplicate in 96-well plates at a cell density of 1 × 10^4^ cells/well and cultured for 24 h prior to treatment with Tg (Sigma-Aldrich, Zwijndrecht, Netherlands) or Tm (Merck Millipore, Amsterdam, the Netherlands) at indicated concentrations and time periods. After treatment, cell viability was determined using MTS assay by incubation with 3-(4,5-dimethylthiazol-2-yl)-5-(3-carboxymethoxyphenyl)-2-(4-sulfophenyl)-2H-tetrazolium solution according to manufacturer’s instructions (Promega Corporation, Leiden, Netherlands). Cell viability was determined by measuring the absorption at 492 nm on a Microplate reader (BioRad, Veenendaal, Netherlands). When indicated cells were (pre)treated with the following chemicals: caspase inhibitor Z-VAD (Promega Corporation, Leiden, Netherlands), RIPK1 inhibitor necrostatin-1 and IRE1α inhibitor 4µ8c (Axon Medchem, Groningen, Netherlands), PERK inhibitors GSK2606414 and AMG PERK 44, GADD34 inhibitor Guanabenz acetate (Tocris Bioscience, Bristol, UK and eIF2α inhibitor ISRIB (Sigma-Aldrich, Zwijndrecht, Netherlands). Caspase-3/7 activities were measured by using the Caspase-Glo 3/7 Caspase-Glo® 3/7 Assay kit (Promega Corporation, Leiden, Netherlands) following the manufacturer’s instructions. Cells were seeded in triplicate on a white 96-well plate at a cell density of 1 × 10^4^ cells/well and pre-cultured for 24 h before treatment with the ER stress inducing drugs at the indicated drug concentrations for the given time period.

### Western blotting

Western blotting was performed as described previously^47^. Standard treatment of cells was 24 h with Tg IC_50_ concentrations unless otherwise stated. The membranes were incubated overnight with the indicated primary antibody (see Supplementary Table 3). Quantified bands are shown in Supplementary Fig. 6.

### RNA isolation and qRT-PCR

Cells were treated as indicated and RNA was isolated from cell pellets using TRIzol® Reagent (Life Technologies, Thermo Fisher Scientific, Bleiswijk, Netherlands) following the manufacturer’s protocol. RNA quantification and cDNA synthesis for qRT-PCR was performed as previously described^46^. Briefly, qRT-PCR was performed in triplicate using the iTaq SYBR Green Supermix with Rox dye (BioRad, Veenendaal, Netherlands) in CFX384 Touch^TM^ Real-Time PCR Detection System C1000 Thermocycler (BioRad, Veenendaal, Netherlands). Amplification was performed with the following cycling conditions: 5 min at 95 °C and 40 two-step cycles of 5 s at 95 °C and 30 s at 60 °C. Cycle threshold (Ct) values for individual reactions were obtained using CFX Manager Software (BioRad, Veenendaal, Netherlands). To determine relative gene expression levels, the C_T_ values were normalized to the house-keeping gene GAPDH using the ΔCt method. Human primers used were previously reported^29^.

### mRNA sequencing and analyses

Illumina next-generation sequencing was performed by the Genome Analysis Facility (GAF), Genomics Coordination Centre (GCC) at University Medical Centre Groningen (Groningen, Netherlands). Initial quality check of and RNA quantification of the samples was performed by capillary electrophoresis using the LabChip GX (PerkinElmer, Groningen, Netherlands). Non-degraded RNA-samples were selected for subsequent sequencing analysis. Sequence libraries were generated using the TruSeq RNA sample preparation kits (Illumina) using the Sciclone NGS Liquid Handler (PerkinElmer, Groningen, Netherlands). In case of contamination of adapter duplexes an extra purification of the libraries was performed with the automated agarose gel separation system Labchip XT (PerkinElmer, Groningen, Netherlands). The obtained cDNA fragment libraries were sequenced on an Illumina HiSeq2500 using default parameters (single read 1 × 50 bp or paired-end 2 × 100 bp) in pools of multiple samples. Sequenced reads were trimmed and subsequently aligned to build b37 human reference genome using HISAT2 0.1.5^48^ and SAMtools 1.2^49^ allowing for two mismatches. Gene level quantification was done using HTSeq/0.6.1p1^50^ using --mode = union--stranded = no. Ensembl v75 was used as reference for gene annotation. Genes with <40 reads were kept out of the analysis. Then reads counts were normalized using trimmed mean of the M-values method. Differential expression (DE) analysis between conditions was done using the DESeq2 package^51^ for R (http://www.r-project.org/). Each DE analysis was performed using paired samples and including library size as covariate. Genes that had an adjusted *p*-value ≤ 0.05, and a Log2 Fold Change more or less than 1 were define as significant differentially expressed genes (DEG).

### Limiting dilution assay

Cells untreated or treated as indicated were pelleted and washed with PBS followed by Accutase (Sigma-Aldrich, Zwijndrecht, Netherlands) treatment and careful repeated pipetting in medium to dissociate cells. The single cell suspension was sorted based on forward and side scatter pattern using a flow cytometer (BD Biosciences, Breda, Netherlands). Single cells were seeded in 96-well plates at a density of 10, 20, 40, or 80 cells/well in a volume of 100 μl NSM; cells were replenished with 50 μl of NSM every 5–7 days. After 3 weeks, the number of neurospheres per well was counted. Each condition was performed in duplicate.

### Generation of CRISPR/Cas9 knockouts

crRNAs were designed using https://benchling.com. DNA oligonucleotides for PERK (EIF2AK3_guide_exon_1_1_FWD CACCGAGACAGAGTTGCGACCGCG and EIF2AK3_guide_exon_1_1_REV aaacCGCGGTCGCAACTCTGTCTC) and ATF4 (ATF4_exon_1_1_FWD CACCGAGGTCTCTTAGATGATTACC and ATF4_exon_1_1_REV aaacGGTAATCATCTAAGAGACCTC) were ordered from IDT (Leuven, Belgium) and cloned into pSpCas9(BB)-2A-GFP(PX458) (Addgene Teddington, UK), following the published protocol by Ann Ran et al.^52^. After transformation in bacteria (One Shot™ TOP10 Chemically Competent *E. coli*; Thermo Fisher Scientific, Bleiswijk, Netherlands), successful cloning was validated by sequencing. GG16 cells were transfected using FuGENE® HD Transfection Reagent (Promega Corporation, Leiden, Netherlands) following manufacturer protocol. After 48 h cells were dissociated and single cell sorting for GFP positivity in 96-well plates. Cells were replenished with 50 μl of NSM twice weekly, and after 3–4 weeks, neurospheres were passaged to a 48-well plate for expansion. Effective ablation of PERK and ATF4 was analyzed by western blotting. Representative *PERK* and *ATF4* knockouts (ko) were selected together with control transfected GG16 cells for further use.

### Immunofluorescence

Cells on cytospins were fixed with 3.7% formaldehyde-PBS followed by permeabilization with 0.1% Triton-X 100-PBS. After blocking with 2% BSA/0.1% Tween20/normal goat serum/PBS primary mouse anti SOX2 antibody (MAB2018, R&D Systems, Bristol, UK) and the corresponding goat-anti-mouse secondary antibody Alexa488 labeled (Life Technologies, Thermo Fisher Scientific, Bleiswijk, Netherlands) were applied. DAPI was used to counterstain nuclei and slides were mounted with Glycerol/Gelatin solution. Fluorescent images of the staining were visualized by fluorescence microscopy (Leica DM-6000 Microscope; Wetzlar, Germany) and images of each condition were captured.

### Statistical analysis

All experiments were performed for at least three times independently unless otherwise stated. Statistical analysis was performed using double sided, paired or unpaired (depending on conditions) Student *t*-test. A *p*-value < 0.05 was considered significant. Statistics used for IHC and RNA-seq analysis are described in the corresponding section.

## Supplementary information

Supplementary material

Supplementary Figures
