# Supplementary material for: ER stress and UPR activation in glioblastoma: identification of a noncanonical PERK mechanism regulating GBM stem cells through SOX2 modulation
Source: Cell Death Dis. 2019 Sep 18;10(10):690. doi: 10.1038/s41419-019-1934-1 (PMC6751174; doi:10.1038/s41419-019-1934-1)
Supplement: Supplementary file 1 — Supplementary material [file 41419_2019_1934_MOESM1_ESM.docx]

**Supplementary Information**

**Supplementary Methods**

**TMA, Immunohistochemistry and analysis**

Before application of the antibodies on the TMA sections, test runs were performed on whole slides of GBM as well as normal brain in order to optimize antibody dilutions and whether antigen retrieval was required to obtain optimal specificity. The TMA was incubated overnight at 60 °C. Tissue sections were deparaffinized and antigen retrieval was performed depending on the antibodies used (see Supplementary Table 2 for antibodies used). After blocking of the endogenous peroxidase, sections were incubated with the primary antibody in 1% BSA/PBS for 1 h at RT, followed by incubation with HRP-conjugated secondary and tertiary antibodies (both Dako-Agilent, Amstelveen, Netherlands) in 1% BSA/PBS with 1% AB serum, each for 30 min at RT. Staining was visualized by 3,3’-diaminobenzidine, followed by counterstaining with haematoxylin. Finally sections were mounted with Eukitt® Quick-hardening mounting medium (Sigma-Aldrich, Zwijndrecht, Netherlands).

To optimize automated scoring in tumor cells using the positive pixel count algorithm and software of Aperio Image Scope 12.3.3 (Leica Biosystems, Amsterdam, Netherlands), each core was inspected for core integrity, blood vessels, necrotic areas or staining artifacts, which were excluded from the scoring area. For each marker the software scoring parameters were tailored to the correct observed staining pattern cytoplasmic for BiP/GRP78 and nuclear for ATF4. Protein expression was defined as the count of positive pixels plus twice the count of strong positive pixels divided by the scored area of the core evaluated. This resulted in a score ranging from 0.5-11.4 for BiP/GRP78 and 0.4-10.7 for ATF4. For evaluation of the BiP/GRP78 staining the score was divided into two groups according to the median: low staining (0.5–3.9), and moderate-high staining (4.0–11.4). In case of ATF4 staining the score was divided into two groups according to the median: low staining (0.4–3.1), and moderate-high staining (3.2–10.7).

XBP1 scoring was performed by two independent observers (NP and CM) blinded for patient outcome and random samples were validated by a blinded expert pathologist (WD). Protein expression of XBP1 was determined by scoring nuclear staining intensity and percentage of positive cells in each core, presented as Immunoreactive Score (IRS). The percentage staining was scored into 4 categories: (0) no staining, (1) 1% to <10%, (2) 10% to <50%, (3) 50% to <100% positive cells. Intensity was scored in 3 categories as (0) negative, (1) low, (2.5) moderate-high. The IRS was calculated by multiplying the percentage of positive cells with the intensity score, resulting in a score from 1 to 7.5. For evaluation of XBP1 staining the IRS was divided into two groups according to the median: low staining (IRS 0–5.0), and moderate-high staining (IRS 5.1–7.5).

**Lentiviral shRNA mediated silencing of ATF6**

GG16 cells were pelleted and washed with PBS followed by accutase (Sigma-Aldrich, Zwijndrecht, Netherlands) treatment and repeated pipetting in medium to dissociate cells. Cells were seeded in duplicate in a 6-well plate at a cell density of 500 x 10^3^ cells/well. Subsequently 20 μl of ATF-6α shRNA Lentiviral Particles (sc-37699-V) or Control shRNA Lentiviral Particles-A (sc-108080) and 5 μg/ml of Polybrene® (sc-134220) were added (Santa Cruz Biotechnology Inc., Bio-Connect BV, Huissen, Netherlands). After 24 h incubation cells were washed with PBS plus 10% FCS once and two times with PBS, followed by culturing in new medium with 0.5 μg/ml of Puromycin for selection. After selection, cells were maintained in 0.2 μg/ml puromycin in NSM and expanded for experiments.

**Supplementary Tables**

| **Supplementary Table 1**. Correlation between patient characteristics and expression of UPR biomarkers | | | | | | |
| --- | --- | --- | --- | --- | --- | --- |
|  |  | **Age** | **OS** | **GRP78 score** | **ATF4 score** | **XBP1 score** |
| **Age** | r^2^ | 1,0 | **-0,384** | -0,077 | 0,048 | -0,123 |
|  | p.value | - | **0,000*** | 0,354 | 0,566 | 0,151 |
|  | N | 148 | **142** | 147 | 144 | 137 |
| **OS** | r^2^ |  | 1,0 | 0,005 | 0,020 | 0,026 |
|  | p.value |  | - | 0,953 | 0,819 | 0,765 |
|  | N |  | 142 | 141 | 138 | 131 |
| **BIP/GRP78 score** | r^2^ |  |  | 1,0 | **0,217** | 0,121 |
|  | p.value |  |  | - | **0,009*** | 0,161 |
|  | N |  |  | 147 | **143** | 136 |
| **ATF4 score** | r^2^ |  |  |  | 1,0 | **0,203** |
|  | p.value |  |  |  | - | **0,017*** |
|  | N |  |  |  | 144 | **137** |
| **XBP1 score** | r^2^ |  |  |  |  | 1,0 |
|  | p.value |  |  |  |  | - |
|  | N |  |  |  |  | 137 |
| OS= overall survival; Spearman’s correlation test, * p < 0,05, significant | | | | | | |

| Supplementary Table 2. Primary antibodies and antigen retrieval methods used for IHC staining | | | | |
| --- | --- | --- | --- | --- |
| Antibody | **Origin** | **Dilution** | **Antigen Retrieval** | **Source** |
| BiP/GRP78 | Rabbit | 1:1600 | Citrate buffer, pH 6.0 | ab21685* |
| XBP1 | Rabbit | 1:1600 | Citrate buffer pH 6.0 | ab109221* |
| ATF4 | Rabbit | 1:1600 | Tris/EDTA buffer pH 9.0 | ab184909* |
| *Abcam, ITK diagnostics BV, Uithoorn, Netherlands | | | | |

| Supplementary Table 3. Primary antibodies for protein detection by western blot | | | |
| --- | --- | --- | --- |
| Antibody | **Origin** | **Source** | **Company** |
| β-actin | Mouse | 69100 | ICN Biomedicals, Zoetermeer, Netherlands |
| ATF4 | Rabbit | ab184909 | Abcam, ITK diagnostics BV, Uithoorn, Netherlands |
| ATF6 | Mouse | ab11909 | Abcam, ITK diagnostics BV, Uithoorn, Netherlands |
| BiP/GRP78 | Rabbit | ab21685 | Abcam, ITK diagnostics BV, Uithoorn, Netherlands |
| Caspase/cleaved caspase 3 | Rabbit | #9662 | Cell Signaling Technology, Bioke, Leiden, Netherlands |
| CHOP | Mouse | #2895 | Cell Signaling Technology, Bioke, Leiden, Netherlands |
| eIF2α | Mouse | #2103 | Cell Signaling Technology, Bioke, Leiden, Netherlands |
| GFAP | Mouse | #3670 | Cell Signaling Technology, Bioke, Leiden, Netherlands |
| Nestin | Mouse | sc-23927 | Santa Cruz Biotechnology Inc., Bio-Connect BV, Huissen, Netherlands |
| Oct4 | Rabbit | #2750 | Cell Signaling Technology, Bioke, Leiden, Netherlands |
| Olig2 | Rabbit | 18953 | Tecan Benelux, Giessen, Netherlands |
| PARP | Rabbit | #9532 | Cell Signaling Technology, Bioke, Leiden, Netherlands |
| PERK and p-PERK | Rabbit | #5683 | Cell Signaling Technology, Bioke, Leiden, Netherlands |
| Phospho-eIF2α | Rabbit | #3398 | Cell Signaling Technology, Bioke, Leiden, Netherlands |
| SOX2 | Mouse | MAB2018 | R&D Systems, Bristol, UK |
| XBP1-unspliced and spliced isoform | Goat | sc-32135 | Santa Cruz Biotechnology Inc., Bio-Connect BV, Huissen, Netherlands |
