# Supplementary material for: ER stress and UPR activation in glioblastoma: identification of a noncanonical PERK mechanism regulating GBM stem cells through SOX2 modulation
Source: Cell Death Dis. 2019 Sep 18;10(10):690. doi: 10.1038/s41419-019-1934-1 (PMC6751174; doi:10.1038/s41419-019-1934-1)
Supplement: Supplementary file 2 — Supplementary Figures [file 41419_2019_1934_MOESM2_ESM.pptx]

## Slide 1
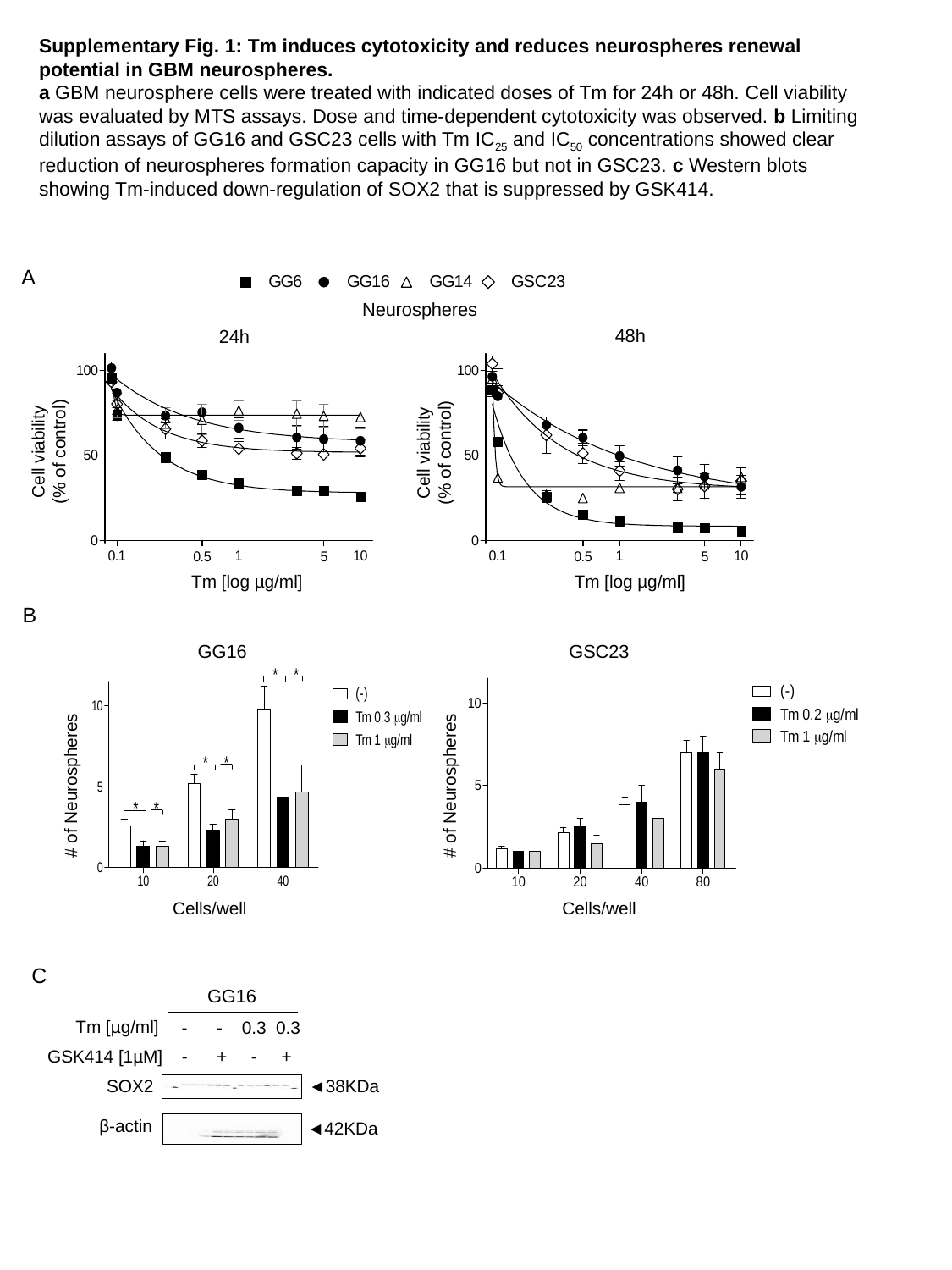

Supplementary Fig. 1: Tm induces cytotoxicity and reduces neurospheres renewal potential in GBM neurospheres.
a GBM neurosphere cells were treated with indicated doses of Tm for 24h or 48h. Cell viability was evaluated by MTS assays. Dose and time-dependent cytotoxicity was observed. b Limiting dilution assays of GG16 and GSC23 cells with Tm IC25 and IC50 concentrations showed clear reduction of neurospheres formation capacity in GG16 but not in GSC23. c Western blots showing Tm-induced down-regulation of SOX2 that is suppressed by GSK414.
A
Neurospheres
48h
24h
Cell viability
(% of control)
Cell viability
(% of control)
Tm [log µg/ml]
Tm [log µg/ml]
B
GG16
GSC23
# of Neurospheres
# of Neurospheres
Cells/well
Cells/well
C
GG16
Tm [µg/ml]
 - - 0.3 0.3
GSK414 [1µM]
 - + - +
◄38KDa
SOX2
β-actin
◄42KDa

## Slide 2
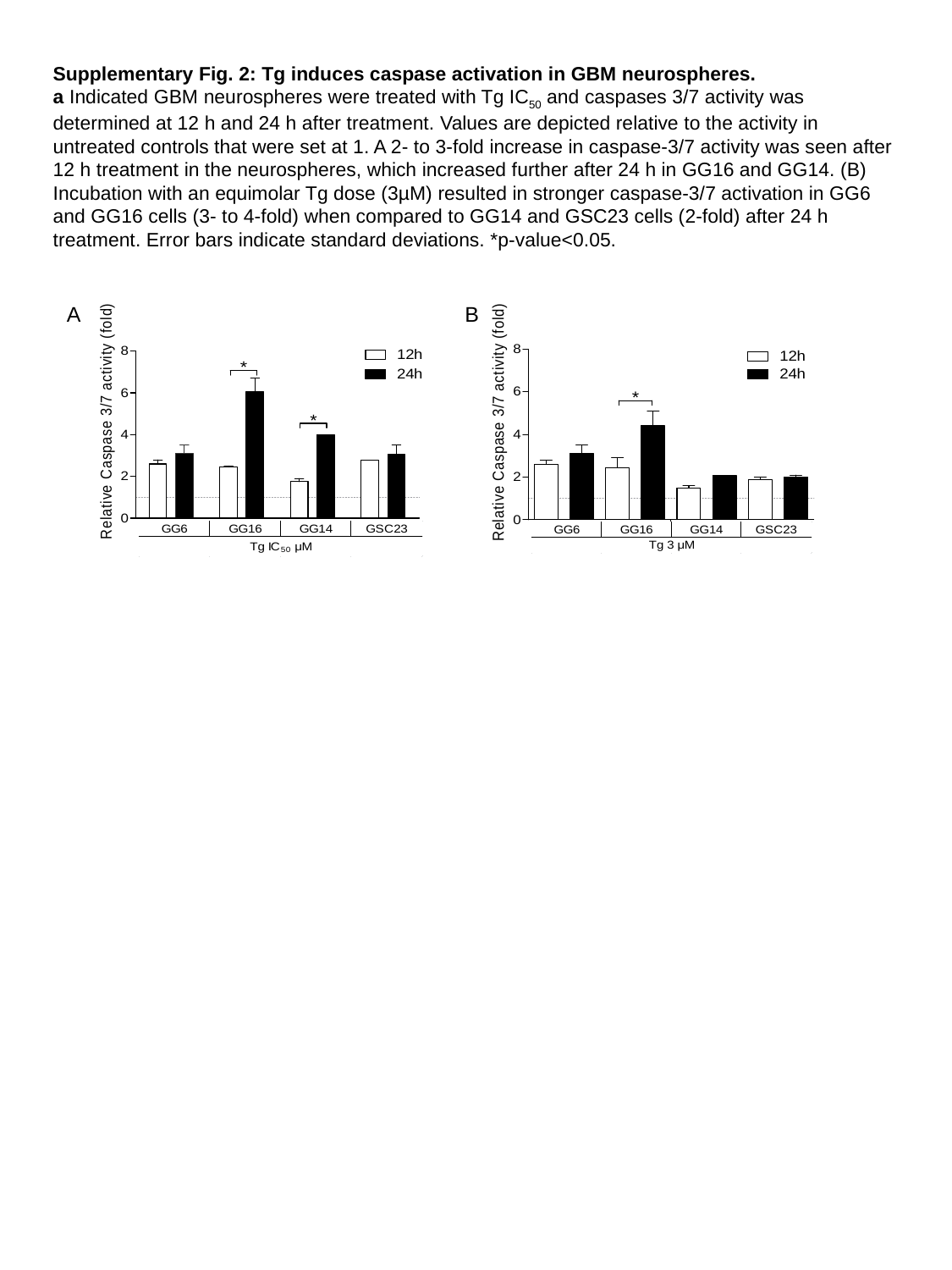

Supplementary Fig. 2: Tg induces caspase activation in GBM neurospheres.
a Indicated GBM neurospheres were treated with Tg IC50 and caspases 3/7 activity was determined at 12 h and 24 h after treatment. Values are depicted relative to the activity in untreated controls that were set at 1. A 2- to 3-fold increase in caspase-3/7 activity was seen after 12 h treatment in the neurospheres, which increased further after 24 h in GG16 and GG14. (B) Incubation with an equimolar Tg dose (3µM) resulted in stronger caspase-3/7 activation in GG6 and GG16 cells (3- to 4-fold) when compared to GG14 and GSC23 cells (2-fold) after 24 h treatment. Error bars indicate standard deviations. *p-value<0.05.
A
B

## Slide 3
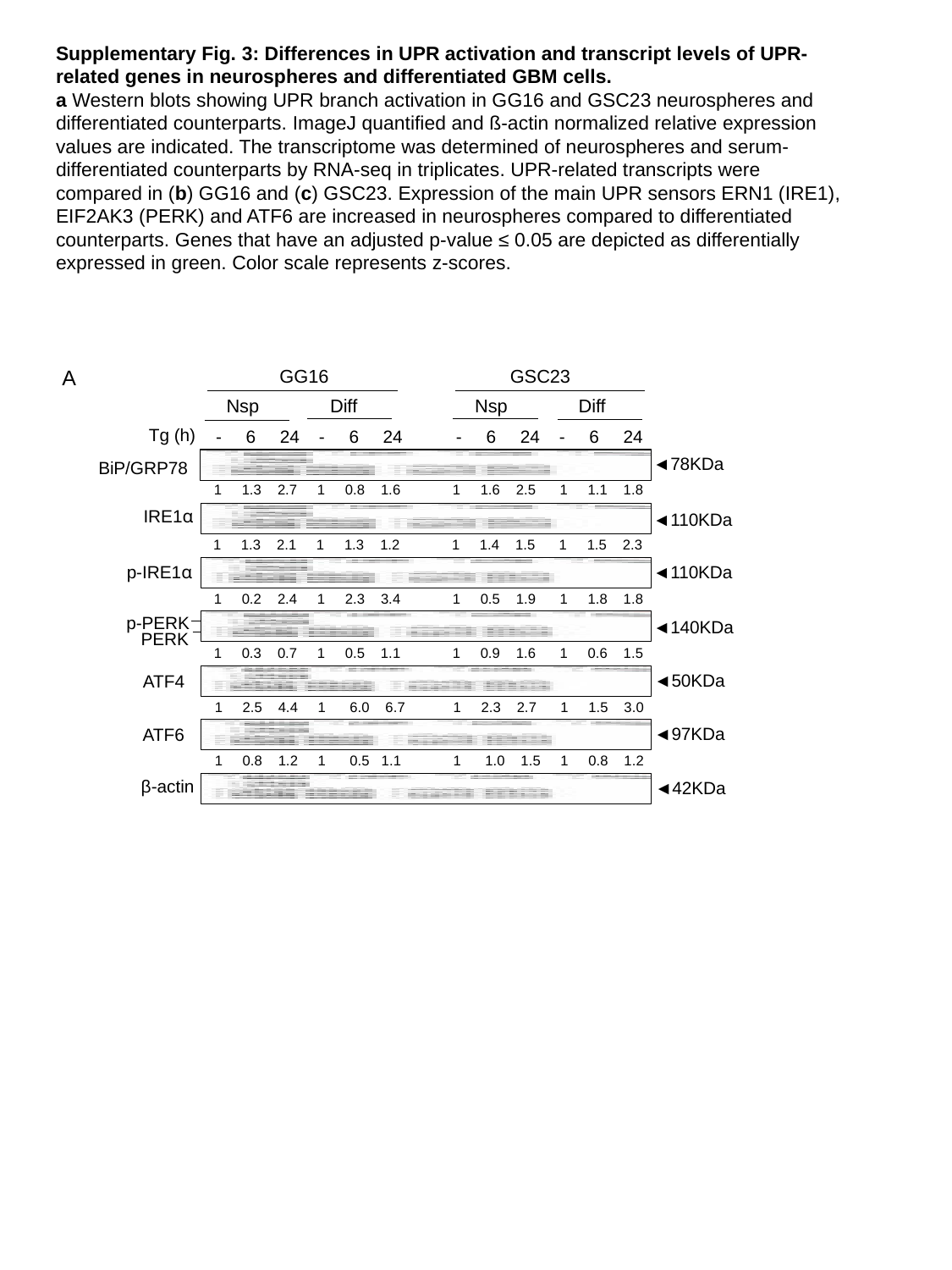

Supplementary Fig. 3: Differences in UPR activation and transcript levels of UPR-related genes in neurospheres and differentiated GBM cells.
a Western blots showing UPR branch activation in GG16 and GSC23 neurospheres and differentiated counterparts. ImageJ quantified and ß-actin normalized relative expression values are indicated. The transcriptome was determined of neurospheres and serum-differentiated counterparts by RNA-seq in triplicates. UPR-related transcripts were compared in (b) GG16 and (c) GSC23. Expression of the main UPR sensors ERN1 (IRE1), EIF2AK3 (PERK) and ATF6 are increased in neurospheres compared to differentiated counterparts. Genes that have an adjusted p-value ≤ 0.05 are depicted as differentially expressed in green. Color scale represents z-scores.
GSC23
A
GG16
 Nsp Diff
 Nsp Diff
Tg (h)
 - 6 24 - 6 24 - 6 24 - 6 24
◄78KDa
BiP/GRP78
1 1.3 2.7 1 0.8 1.6 1 1.6 2.5 1 1.1 1.8
IRE1α
◄110KDa
1 1.3 2.1 1 1.3 1.2 1 1.4 1.5 1 1.5 2.3
◄110KDa
p-IRE1α
1 0.2 2.4 1 2.3 3.4 1 0.5 1.9 1 1.8 1.8
p-PERK
◄140KDa
PERK
1 0.3 0.7 1 0.5 1.1 1 0.9 1.6 1 0.6 1.5
◄50KDa
ATF4
1 2.5 4.4 1 6.0 6.7 1 2.3 2.7 1 1.5 3.0
◄97KDa
ATF6
1 0.8 1.2 1 0.5 1.1 1 1.0 1.5 1 0.8 1.2
β-actin
◄42KDa

## Slide 4
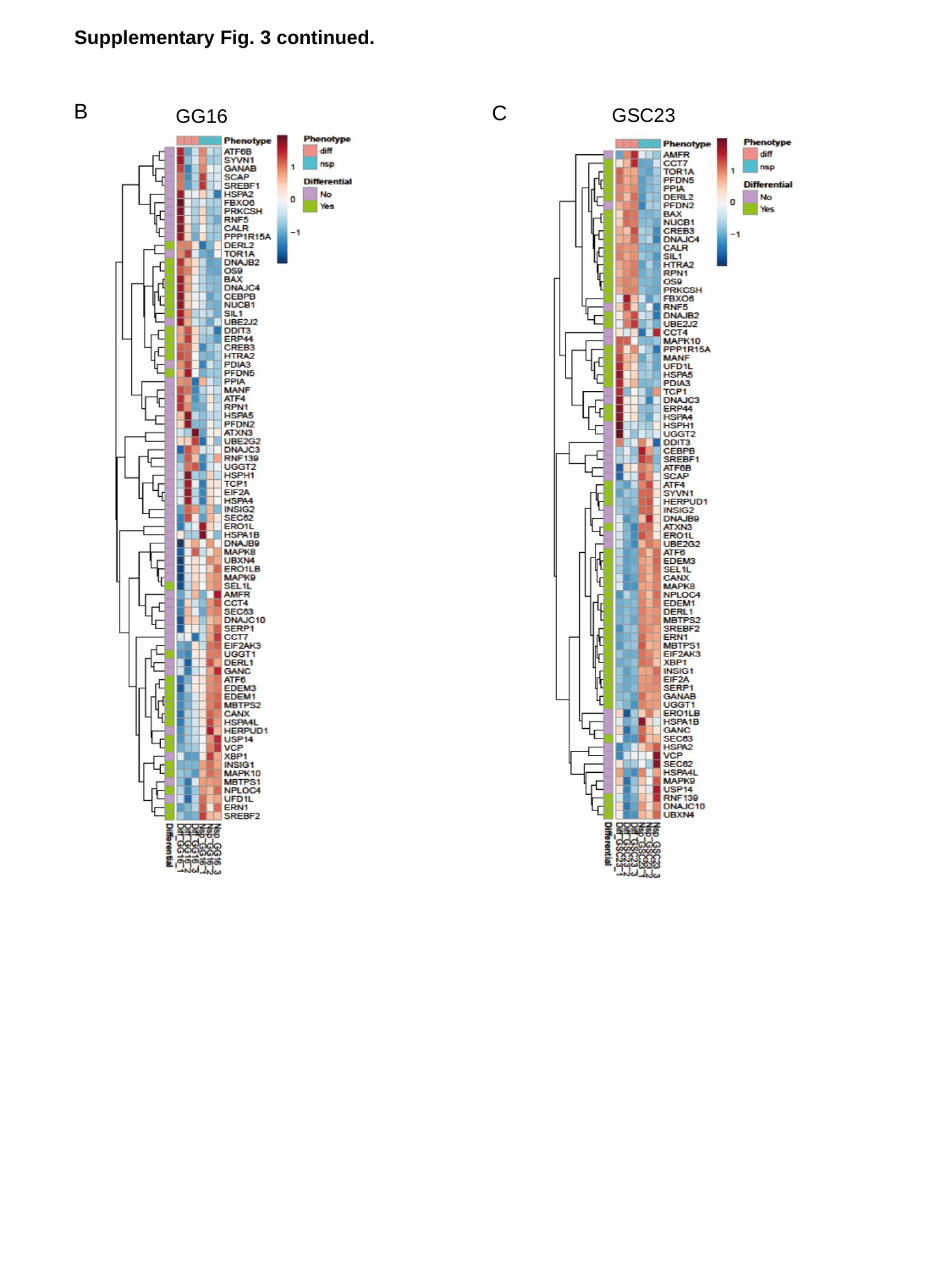

Supplementary Fig. 3 continued.
B
C
GSC23
GG16

## Slide 5
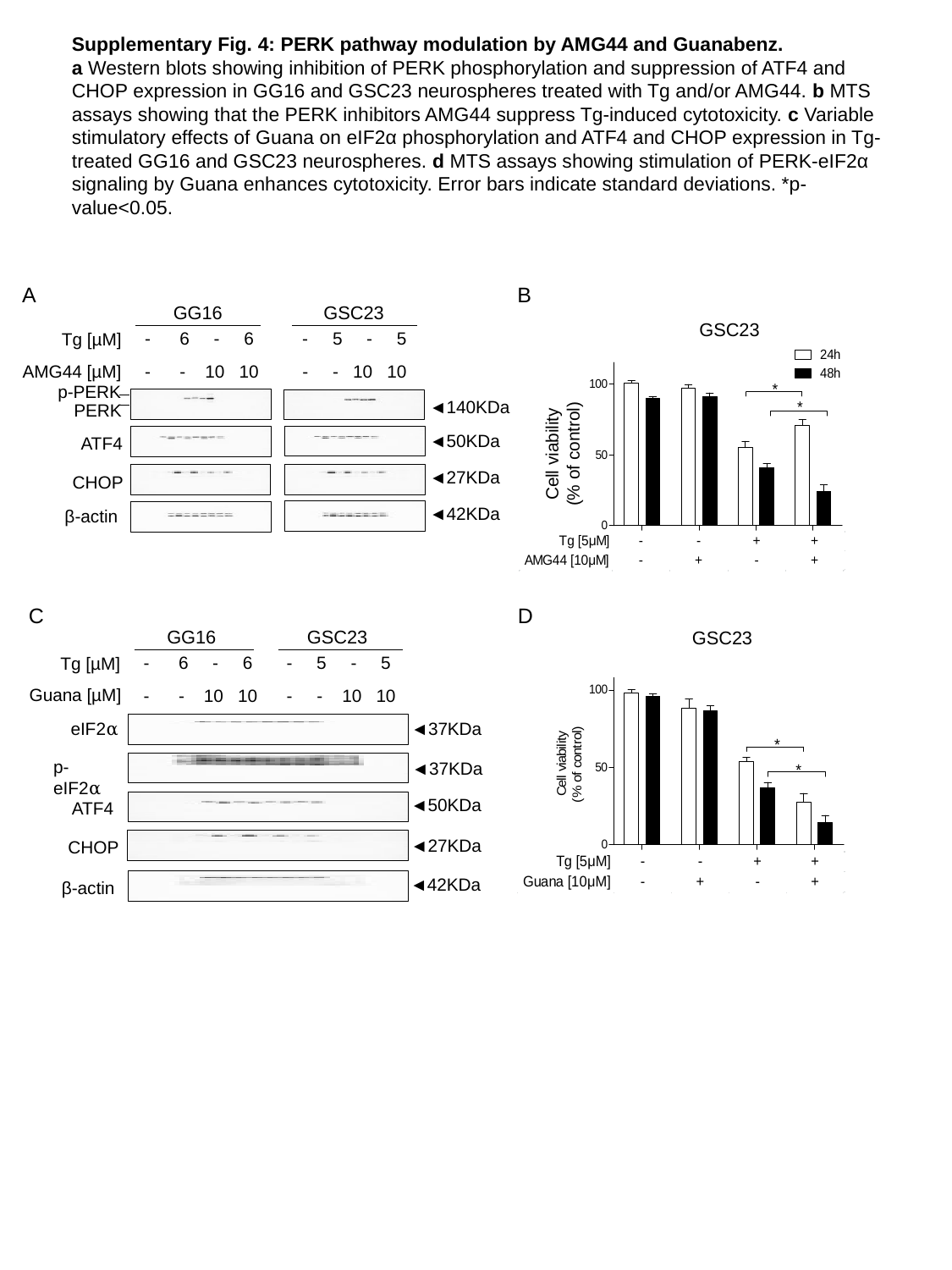

Supplementary Fig. 4: PERK pathway modulation by AMG44 and Guanabenz.
a Western blots showing inhibition of PERK phosphorylation and suppression of ATF4 and CHOP expression in GG16 and GSC23 neurospheres treated with Tg and/or AMG44. b MTS assays showing that the PERK inhibitors AMG44 suppress Tg-induced cytotoxicity. c Variable stimulatory effects of Guana on eIF2α phosphorylation and ATF4 and CHOP expression in Tg-treated GG16 and GSC23 neurospheres. d MTS assays showing stimulation of PERK-eIF2α signaling by Guana enhances cytotoxicity. Error bars indicate standard deviations. *p-value<0.05.
B
A
 GG16 GSC23
 - 6 - 6 - 5 - 5
Tg [µM]
AMG44 [µM]
 - - 10 10 - - 10 10
p-PERK
PERK
ATF4
CHOP
β-actin
GSC23
◄140KDa
◄50KDa
Cell viability
(% of control)
◄27KDa
◄42KDa
C
D
 GG16 GSC23
 - 6 - 6 - 5 - 5
Tg [µM]
Guana [µM]
 - - 10 10 - - 10 10
eIF2α
p-eIF2α
ATF4
CHOP
β-actin
GSC23
◄37KDa
◄37KDa
◄50KDa
◄27KDa
◄42KDa

## Slide 6
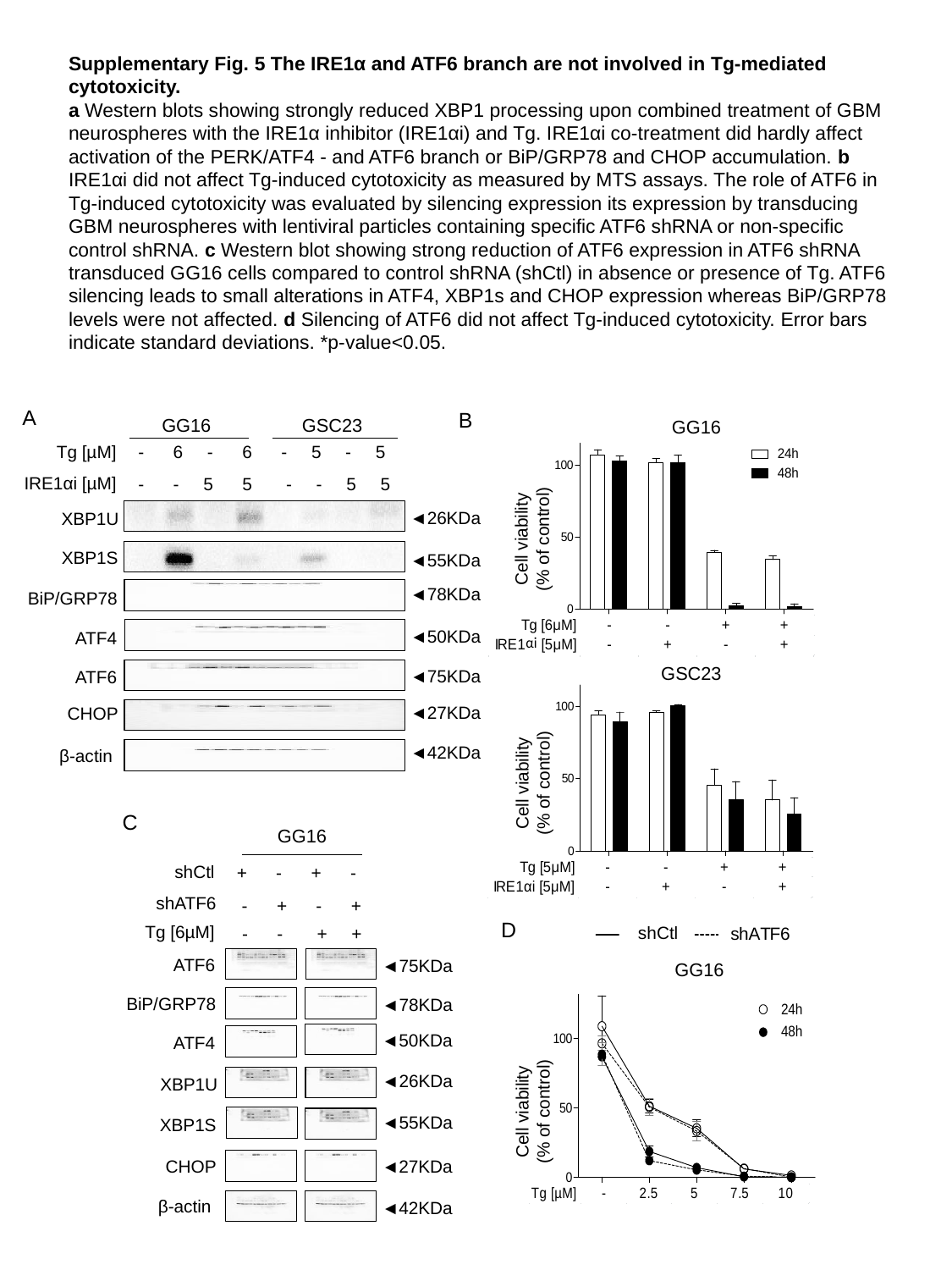

Supplementary Fig. 5 The IRE1α and ATF6 branch are not involved in Tg-mediated cytotoxicity.
a Western blots showing strongly reduced XBP1 processing upon combined treatment of GBM neurospheres with the IRE1α inhibitor (IRE1αi) and Tg. IRE1αi co-treatment did hardly affect activation of the PERK/ATF4 - and ATF6 branch or BiP/GRP78 and CHOP accumulation. b IRE1αi did not affect Tg-induced cytotoxicity as measured by MTS assays. The role of ATF6 in Tg-induced cytotoxicity was evaluated by silencing expression its expression by transducing GBM neurospheres with lentiviral particles containing specific ATF6 shRNA or non-specific control shRNA. c Western blot showing strong reduction of ATF6 expression in ATF6 shRNA transduced GG16 cells compared to control shRNA (shCtl) in absence or presence of Tg. ATF6 silencing leads to small alterations in ATF4, XBP1s and CHOP expression whereas BiP/GRP78 levels were not affected. d Silencing of ATF6 did not affect Tg-induced cytotoxicity. Error bars indicate standard deviations. *p-value<0.05.
A
B
 GG16 GSC23
GG16
 - 6 - 6 - 5 - 5
Tg [µM]
IRE1αi [µM]
 - - 5 5 - - 5 5
◄26KDa
XBP1U
Cell viability
(% of control)
XBP1S
◄55KDa
◄78KDa
BiP/GRP78
◄50KDa
ATF4
GSC23
◄75KDa
ATF6
◄27KDa
CHOP
◄42KDa
β-actin
Cell viability
(% of control)
C
GG16
shCtl
+ - + -
shATF6
 - + - +
Tg [6µM]
 - - + +
ATF6
BiP/GRP78
ATF4
XBP1U
XBP1S
CHOP
β-actin
D
shCtl
◄75KDa
GG16
◄78KDa
◄50KDa
◄26KDa
Cell viability
(% of control)
◄55KDa
◄27KDa
◄42KDa

## Slide 7
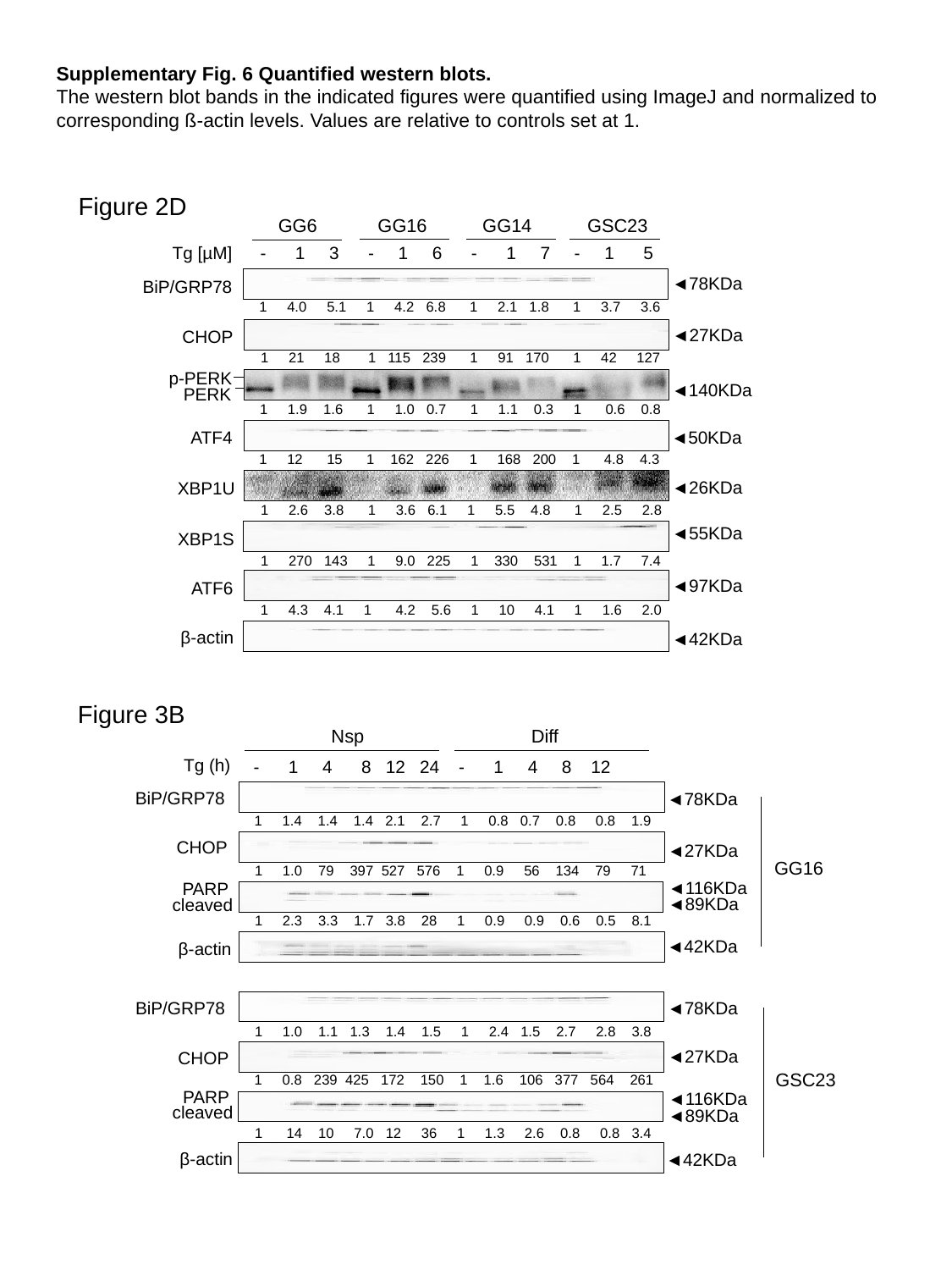

Supplementary Fig. 6 Quantified western blots.
The western blot bands in the indicated figures were quantified using ImageJ and normalized to corresponding ß-actin levels. Values are relative to controls set at 1.
Figure 2D
 GG6 GG16 GG14 GSC23
Tg [µM]
 - 1 3 - 1 6 - 1 7 - 1 5
◄78KDa
BiP/GRP78
 1 4.0 5.1 1 4.2 6.8 1 2.1 1.8 1 3.7 3.6
◄27KDa
CHOP
 1 21 18 1 115 239 1 91 170 1 42 127
p-PERK
◄140KDa
PERK
 1 1.9 1.6 1 1.0 0.7 1 1.1 0.3 1 0.6 0.8
ATF4
◄50KDa
 1 12 15 1 162 226 1 168 200 1 4.8 4.3
◄26KDa
XBP1U
 1 2.6 3.8 1 3.6 6.1 1 5.5 4.8 1 2.5 2.8
◄55KDa
XBP1S
 1 270 143 1 9.0 225 1 330 531 1 1.7 7.4
◄97KDa
ATF6
 1 4.3 4.1 1 4.2 5.6 1 10 4.1 1 1.6 2.0
β-actin
◄42KDa
Figure 3B
 Nsp Diff
Tg (h)
 - 1 4 8 12 24 - 1 4 8 12 24
BiP/GRP78
◄78KDa
 1 1.4 1.4 1.4 2.1 2.7 1 0.8 0.7 0.8 0.8 1.9
CHOP
◄27KDa
GG16
 1 1.0 79 397 527 576 1 0.9 56 134 79 71
◄116KDa
PARP
◄89KDa
cleaved
 1 2.3 3.3 1.7 3.8 28 1 0.9 0.9 0.6 0.5 8.1
◄42KDa
β-actin
BiP/GRP78
◄78KDa
 1 1.0 1.1 1.3 1.4 1.5 1 2.4 1.5 2.7 2.8 3.8
◄27KDa
CHOP
GSC23
 1 0.8 239 425 172 150 1 1.6 106 377 564 261
PARP
◄116KDa
cleaved
◄89KDa
 1 14 10 7.0 12 36 1 1.3 2.6 0.8 0.8 3.4
β-actin
◄42KDa

## Slide 8
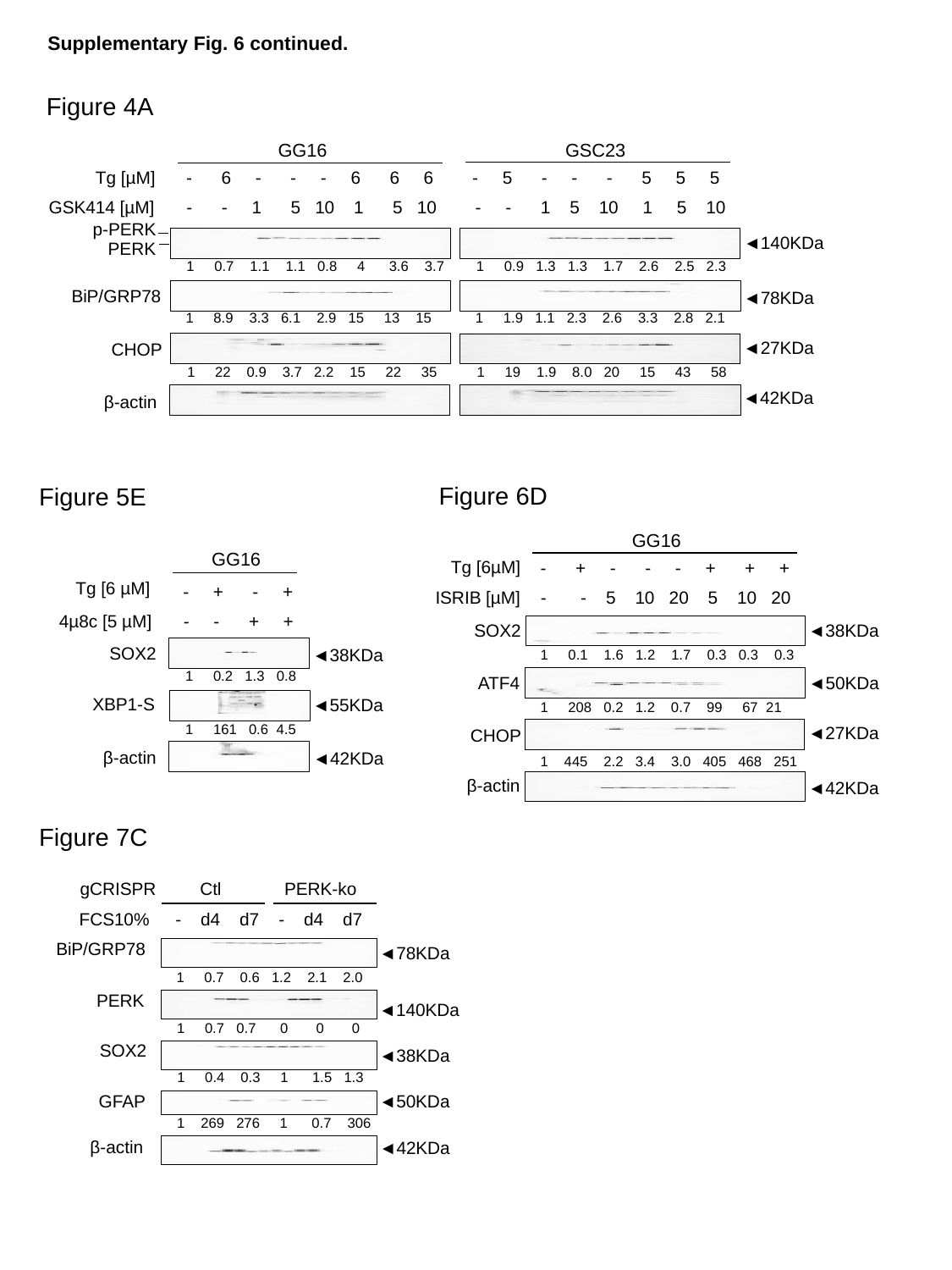

Supplementary Fig. 6 continued.
Figure 4A
 GG16 GSC23
Tg [µM]
 - 6 - - - 6 6 6 - 5 - - - 5 5 5
GSK414 [µM]
 - - 1 5 10 1 5 10 - - 1 5 10 1 5 10
p-PERK
◄140KDa
PERK
 1 0.7 1.1 1.1 0.8 4 3.6 3.7 1 0.9 1.3 1.3 1.7 2.6 2.5 2.3
BiP/GRP78
◄78KDa
 1 8.9 3.3 6.1 2.9 15 13 15 1 1.9 1.1 2.3 2.6 3.3 2.8 2.1
◄27KDa
CHOP
 1 22 0.9 3.7 2.2 15 22 35 1 19 1.9 8.0 20 15 43 58
◄42KDa
β-actin
Figure 6D
Figure 5E
 GG16
GG16
Tg [6µM]
 - + - - - + + +
Tg [6 µM]
 - + - +
ISRIB [µM]
 - - 5 10 20 5 10 20
 - - + +
4µ8c [5 µM]
◄38KDa
SOX2
SOX2
◄38KDa
 1 0.1 1.6 1.2 1.7 0.3 0.3 0.3
 1 0.2 1.3 0.8
◄50KDa
ATF4
XBP1-S
◄55KDa
 1 208 0.2 1.2 0.7 99 67 21
 1 161 0.6 4.5
◄27KDa
CHOP
β-actin
◄42KDa
 1 445 2.2 3.4 3.0 405 468 251
β-actin
◄42KDa
Figure 7C
gCRISPR
 Ctl PERK-ko
FCS10%
 - d4 d7 - d4 d7
BiP/GRP78
◄78KDa
 1 0.7 0.6 1.2 2.1 2.0
PERK
◄140KDa
 1 0.7 0.7 0 0 0
SOX2
◄38KDa
 1 0.4 0.3 1 1.5 1.3
GFAP
◄50KDa
 1 269 276 1 0.7 306
β-actin
◄42KDa
